# Non-negligible Outer-Shell Reorganization Energy for Charge Transfer in Nonpolar Systems

**DOI:** 10.1021/acs.jctc.4c00742

**Published:** 2024-08-15

**Authors:** Chou-Hsun Yang, Chun-I Wang, Yi-Siang Wang, Chao-Ping Hsu

**Affiliations:** † Institute of Chemistry, Academia Sinica, 128 Section 2 Academia Road, Nankang, Taipei 115, Taiwan; ‡ National Center for Theoretical Sciences, 1, Section 4, Roosevelt Road, Taipei 106, Taiwan

## Abstract

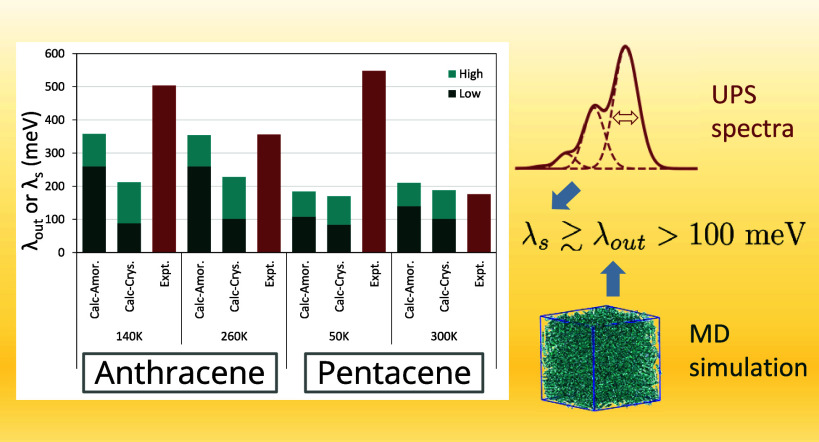

Many charge-transporting molecular systems function as ordered or disordered arrays of solid state materials composed of nonpolar (or weakly polar) molecules. Due to low dielectric constants for nonpolar systems, it is common to ignore the effects of outer-shell reorganization energy (λ_*out*_). However, ignoring λ_*out*_ has not been properly supported and it can severely impact predictions and insights derived. Here, we estimate λ_*out*_ by two means: from experimental ultraviolet photoelectron spectra, in which vibronic progression in these spectra can be fitted with the widths of peaks determining the low-frequency component in reorganization energy, regarded to be closely associated with λ_*out*_, and from molecular dynamic (MD) simulation of nonpolar molecules, in which disorder or fluctuation statistics for energies of charged molecules are calculated. An upper bound for λ_*out*_ was obtained as 505 and 549 meV for crystalline anthracene (140 K) and pentacene (50 K), respectively, by fitting of experimental data, and 212 and 170 meV, respectively, from MD simulations. These values are comparable to the inner-sphere reorganization energy (λ_*in*_) arising from intramolecular vibration. With corresponding spectral density functions calculated, we found that λ_*out*_ is influenced both by low- and high-frequency dynamics, in which the former arises from constrained translational and rotational motions of surrounding molecules. In an amorphous state, about half of the λ_*out*_’s obtained are from high-frequency components, which is quite different from the conventional polar solvation. Moreover, crystalline systems exhibit super-Ohmic spectral density, whereas amorphous systems are sub-Ohmic.

## 1 Introduction

Electron transfer (ET) theory^1^ has been an important foundation for understanding charge transfer processes in many systems. One important application area is in charge-transporting materials, such as organic light-emitting diodes, solar cells, and field-effect transistors, *etc*.^2−8^ With their advantages of low cost, low mass, and flexibility, organic molecular semiconductors have attracted much attention.^9^ In the Marcus theory, ET rate can expressed as
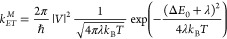
1where *V* is the electronic coupling between initial and final states, and Δ*E*_0_ is the zero-point energy difference of the two states. The reorganization energy λ is a critical factor in the Marcus theory, as it is tightly linked to the activation energy needed for electron transfer. Therefore, molecules with low λ values have become important for systems with good charge transporting behaviors.^10−14^ λ is also useful for finding organic photovoltaics with good charge separation without recombination.^15−17^

λ comprises two components: the inner-sphere λ_*in*_, arising from intramolecular degrees of freedom, and the outer-shell, λ_*out*_, from changes in the surrounding environment. The former can be estimated from standard quantum chemistry calculations^18,19^ in which the energy difference of two reactant and product structures in the same state is calculated, with the electronic state being that for either reactant, or product, in the calculation. The latter, λ_*out*_, can be calculated using a simple expression^1,20^
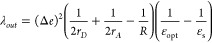
2where the two fragments involved in the ET, the donor *D* and the acceptor *A* are modeled as spherical cavities with radii *r*_*D*_ and *r*_*A*_, with their center-to-center distance *R*, embedded in a dielectric medium characterized by static and optical dielectric constants ε_s_ and ε_opt_, respectively. According to eq 2, nonpolar solvents have very similar ε_s_ and ε_opt_ values, leading to vanishing λ_*out*_, e.g. 20–30 meV for benzothieno-benzothiophene (BTBT) with long alkyl side-chains,^21^ and 33–42 meV for fullerene.^22^ Therefore, it is very common to simply take the calculated λ_*in*_ as the only source of reorganization energy.^18,19,23−26^

Values of λ_*out*_ can be inferred from experiments, and most of them are on the order of 100 meV, similar to many λ_*in*_ values reported. In ref (27)., an experimental work, λ_*out*_ 310 and 140 meV were fitted from data obtained with electron attachment. λ_*out*_ can also be estimated from dynamic Stokes shifts in a number of nonpolar solvents, which can reach 150 meV. Interestingly, a correlation for the Stokes shift value to effective quadrupole moments was observed.^28^ By fitting emission spectra with a semiclassical Marcus equation, the reorganization energy derived from the width of the vibronic progression is largely contributed by λ_*out*_. In a porphyrin-fullerene system in benzene, it was estimated as 130 meV.,^29^ and 80–532 meV for various donors in fullerene-based organic solar cell systems.^30^

Theoretical estimates of λ_*out*_ for nonpolar solvents are seen with various approaches. Based on dipolar solvation theories with structural factors considered,^31,32^ the program SolvMol yielded solvent reorganizations of 2.58 and 0.24 meV for meta- and para-phenyl-acetylene-bridged carbazole-naphthalimide in weakly polar toluene.^33^ With the density structural factor of solvents considered, an expression for the induced component in λ in response to structural changes, λ^*ind*^ was developed,^34^ and it is estimated as 100–300 meV for nonpolar solvents, a range that fits experimental observations. Even though the dipolar contribution does not exist in nonpolar solvents, a work focusing on quadrupole solvation was developed,^35^ and values of 165–205 meV were reported for the reorganization energy of benzene, a range that is similar to the magnitude of commonly reported λ_*in*_ for organic molecules exhibiting good semiconducting potential (In ref (23)., λ_*in*_ values reported are 138 and 102 meV, for tetracene and pentacene, respectively.) Therefore, with dielectric polarization theory, both the density structural factor (structural density fluctuation) in electric induction, and the quadrupole moments of solvent can lead to significant values in λ_*out*_

In the Marcus theory, the reaction coordinate is a collective quantity over many degrees of freedom. Free energy curves Δ*G* of the initial and final states are assumed to be parabolic, where λ is the curvature. Therefore, the width of a thermal distribution gives rise to the reorganization energy. With this approach, values of 10–100 meV were reported for nonpolar systems.^36,37^

On the other hand, λ_*out*_ has frequently been ignored in computational works.^38−46^ This is usually rationalized with the low dielectric constants in nonpolar environments in those studies, together with the expression in eq 2. Works employing molecular modeling, such as hybrid quantum mechanical/molecular mechanical (QM/MM) schemes and polarizable force fields, λ_*out*_ were calculated with optimization in several common organic molecular crystals, but only 0.98–7.81 meV were obtained.^47,48^ In these works, the energy of one of the charged states was calculated at different optimized structures, a typical method for determining λ_*in*_. Doing so for λ_*out*_ by including the surrounding molecules may sound like a reasonable generalization, but statistical contributions were ignored.

While direct comparisons of computational characterizations of charge transporting behaviors with experimental measurements is not entirely feasible yet, we believe that including contributions of λ_*out*_ in theoretical models is important. Therefore, careful examination of the magnitude of λ_*out*_ in typical nonpolar systems is necessary.

In the present work, we estimate λ_*out*_ from experimental data, as well as direct simulation for nonpolar molecules. We studied ethylene, anthracene, and pentacene in their solid states, with ethylene being a small system suitable for benchmarking. Anthracene and pentacene were chosen for direct comparisons with available experimental data.^49−51^ The experimental bandwidth of ultraviolet photoelectron spectra (UPS) was employed to estimate λ_*out*_. MD simulation was used to account for fluctuation in the interaction energy of a cationic molecule embedded in the system, from which λ_*out*_ was calculated. Both results show values larger than 100 meV, values that cannot easily be ignored. We also studied spectral density functions and their breakdown in frequencies, fitting for the Ohmic model. We further discuss the role of λ_*out*_ in ET rates.

## 2 Theory and Method

### 2.1 Extracting Reorganization Energy from Ultraviolet Photoelectron Spectra

In UPS, an incident high-energy photon ionizes a molecule, ejecting an electron. The energy of the cationic state of the system can be inferred from the kinetic energy of the electron ejected. In this way, UPS spectra show transitions from neutral molecules to cations, with possible vibronic progressions. Since it is half of a hole transfer process, these spectra contain information about the reorganization energy of hole transfer in the medium employed in the experiments.

#### 2.1.1 Theoretical Ground

Based on Marcus-Levich-Dogonadze-Jortner (or Jortner’s) theory, for a system with a high-frequency mode, the electron transfer rate can be expressed as^52−54^
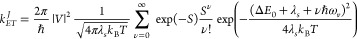
3where *V* is the electronic coupling between initial and final states, and Δ*E*_0_ is the zero-point energy difference of the two states. In this widely recognized formulation, it has been assumed that the reorganization energy can be split into low- and high-frequency contributions, using thermal energy *k*_*B*_*T* as the dividing line. λ_*s*_ is the low-frequency contribution in the reorganization energy, and for the high-frequency contribution, it is usually written with just one representative (effective) mode, with ω_*v*_ being the frequency of the mode, and *S*, the Huang–Rhys (HR) factor, with the high-frequency part of reorganization energy λ_*v*_ as,
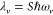
4The original multimode expression can be found at refs (52, 55). Reducing multiple high-frequency modes to an effective mode representing the vibronic progression effect has also been discussed.^52,56^ Multimode with quantum statistical treatment for ET rate has been an active area of theoretical works, and in general, systems without a clear vibronic progression in their density of states can also be treated.^57^ Molecules with strong vibronic progression spectra usually have more than one active, high-frequency mode. As long as they are with similar frequencies, an effective frequency and an effective HR factor can be defined,^56,58^ and eq 4 with such effective factors gives the reorganization energy of these high-frequency modes.

**Figure 1:**
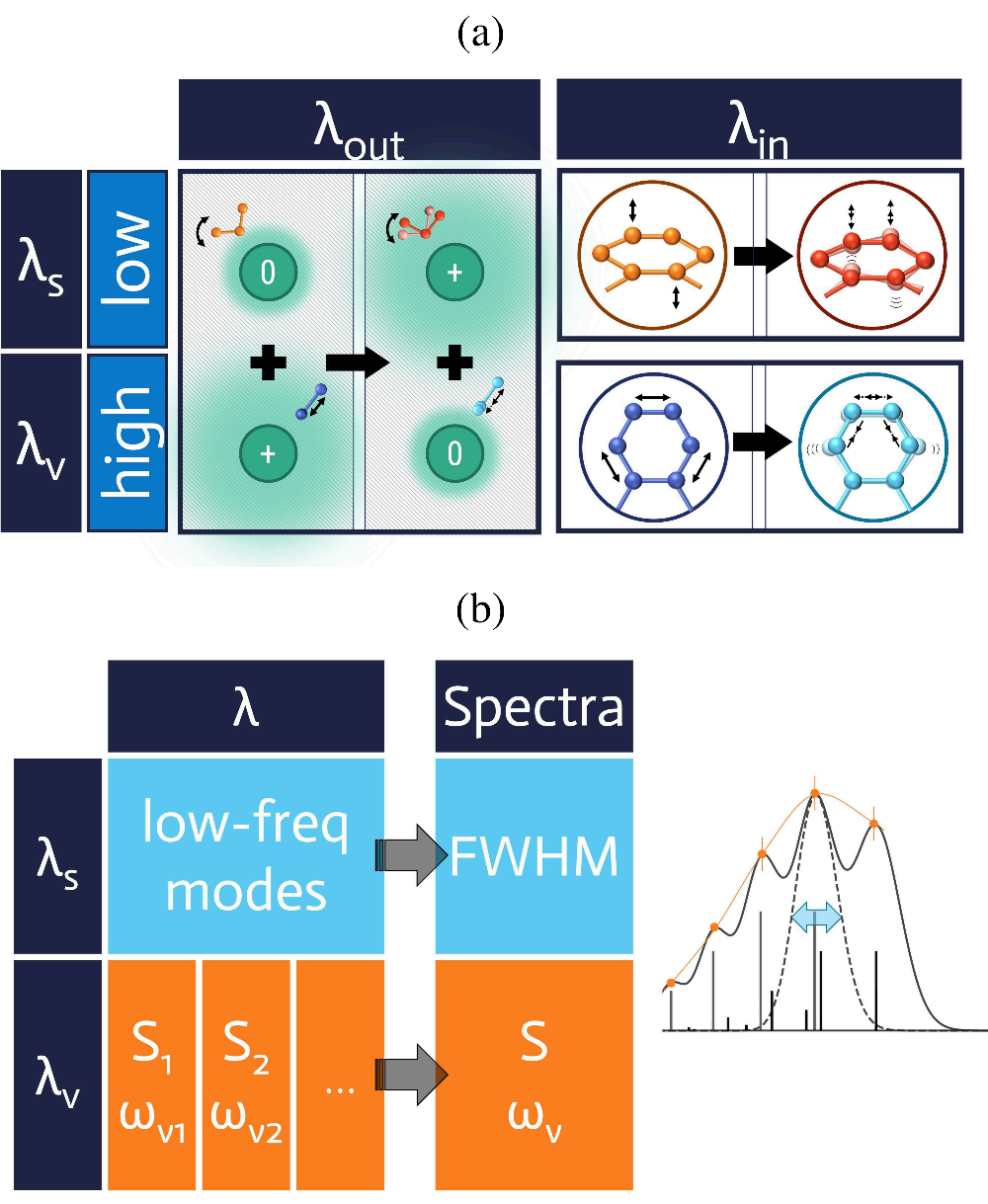
(a) Depiction for different separations of λ: the traditional inner- and outer-shell separation in the right and left columns (λ_*in*_ vs λ_*out*_). The low- and high-frequency separations are depicted in upper and lower rows, which are modeled as λ_*s*_ and λ_*v*_ in fitting photoelectron spectra, as further schematically illustrated in (b).

With vibronic progression in a transition between ionic and neutral states, as in eq 3, the HR factor *S* for a high-frequency vibration that has the largest coupling to the ionization can be inferred.^59^ The width of a UPS spectrum is closely related to λ_*s*_.^49−51,60^ By taking the standard deviation σ of the progressive band, together with Gaussian terms in eq 3, we have
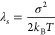
5In practice one can infer the full width at half-maximum (fwhm) easily from experimental spectra, and use the following relationship for a Gaussian function,
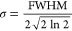
6fwhm can be estimated with fitting to a pseudo-Voigt function, which is a linear combination of Gaussian and Lorentzian profiles.^49−51,60^ Therefore, in the present work, we took the fwhm from experimental literature and used it in the theoretical model composed of Gaussian bands for estimating λ_*s*_eqs 5 and 6 as illustrated in Figure 1.

#### 2.1.2 Partitioning in λ and Possible Discrepancies between λ_*out*_ and λ_*s*_

Illustrative depiction for two different ways to partition the reorganization energy λ are included in Figure 1a. Traditional inner- and outer-shell separation has λ_*out*_ arising from intermolecular interactions, comprised of solute–solvent, or system-bath interaction, and λ_*in*_ mainly arising from intramolecular vibrational degrees of freedom. On the other hand, there exist active high-frequency modes that contribute to the progression of spectra, and the remaining low-frequency modes, and their corresponding portion of reorganization energy are modeled as λ_*s*_ and λ_*v*_ in fitting the photoelectron spectra. Such division allows a simplified classical treatment for the low-frequency mode and the quantum effect of high-frequency modes can be included, leading to Jortner’s rate expression (eq 3) or other multimode variations.^52,56,57^

It is desirable to assume that the two partitionings of λ are highly correlated, i.e., it is likely that the intermolecular interaction is dominated by low-frequency motions, and the reorganization energy from intramolecular vibrational modes is mainly high-frequency,
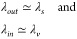
7If so, estimating λ_*s*_ from UPS spectra yields λ_*out*_ directly.

It has been reported that λ_*out*_ mostly consists of low-frequency components.^61−63^ In other words, there might be a high-frequency contribution in λ_*out*_ that belongs to λ_*v*_, and such a contribution is quite small for the polar solvents reported. On the other hand, low-frequency modes, such as ring-distortion, as depicted in Figure 1a, contribute to λ_*in*_ due to its intramolecular nature, but it is possible to contribute to λ_*s*_ with its low frequency. Therefore, rigorously speaking, we have two terms in the discrepancy between λ_*out*_ and λ_*s*_:
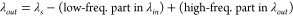
8Another possible discrepancy of this approach is that widths of these progression bands can be broadened by various frequencies of high-frequency modes. The λ_*s*_ from fitting the width of the vibronic bands might be overestimated.

In the present work, we estimated λ_*s*_’s from experimental UPS spectra and cautiously compare them to calculated λ_*out*_. In doing so we simply assumed that these two additional terms in eq 8 are small or they could largely be canceled. Therefore, values of λ_*s*_ from fitting UPS spectra in the literature can be treated as an upper-bound for λ_*out*_. We note that it is not a fully rigorous approach as further details are remained to be explored.

### 2.2 Estimating λ_*out*_ with Molecular Dynamics Simulation

#### 2.2.1 Energetic Disorder and Its Relationship to λ_*out*_

Reorganization energy is the energy required in changing the external coordinates from those in equilibrium with the initial state, to those of the final state, without changing the electronic configuration. For the outer part, λ_*out*_, it is the energy difference arising from system-environment interaction. For example, if we have a hole-transfer event in two identical molecules
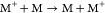
9where we keep the ordering of the first and the second molecules M. The reorganization energy is,
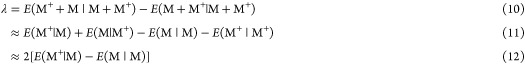
where *E*(*A*|*B*) denotes the (free) energy of state A in the system equilibrated under state B. Since outer-shell reorganization energy involves changes in the environment, in this work, we consider the long-range, electrostatic interaction between the system and surrounding environment. The interaction energy of the additional charge on one molecule and the environment Δ*E*_int_(*t*) is defined as,
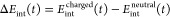
13where *E*_*int*_^neutral^(*t*) is the interaction energy of a target molecule and all other molecules, with the target molecule in its neutral state, and *E*_*int*_^charged^(*t*) being the same interaction energy but with the target molecule in its charged state. After extracting snapshots from MD simulation, we calculate the energy difference of a (arbitrarily chosen) target molecule of its charged and neutral states. In other words, Δ*E*_int_(*t*) is the MD realization of intermolecular, electrostatic contribution of *E*(M^+^|M) – *E*(M|M), whose statistics lead to λ_*out*_.

In principle, twice the ensemble average for Δ*E*_int_ is the outer-shell reorganization energy value we are seeking, λ_*out*_. Since we have MD trajectories, the dynamic aspect of λ_*out*_ can be inferred as well. Equivalently, the reorganization energy can be evaluated using the fluctuation–dissipation theorem.^61,64^ The autocorrelation function (ACF) *C*(*t*) can be defined as
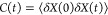
14where *δX*(*t*) ≡Δ*E*_int_(*t*) – ⟨Δ*E*_int_⟩ and ⟨Δ*E*_int_⟩ is the mean value of Δ*E*_int_(*t*) in each trajectory. Therefore, with Fourier transform of ACF  the power spectrum *I*(ω) can be obtained as,
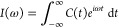
15

In the present work, we employ the spectral density *J*(ω) to describe the fluctuation of electrostatic energies. Under the classical limit, it can be derived from *I*(ω):
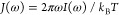
16where we have included a factor of 2 since we were only calculating half of the ET process (M → M^+^) in the simulated Δ*E*_int_(*t*). λ_*out*_ is calculated as,^61,64^
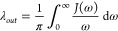
17

To evaluate the quality of theoretical results of λ_*out*_ from MD trajectories, the standard deviation (STD) of the mean value of the autocorrelation function *C*(*t*) is employed. With eqs 14 to (17), and by accounting the factor of 2 in eq 12, λ_*out*_, can also be expressed as,
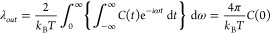
18

In the MD simulation, we include 20,000 ethylene, or 2,000 anthracene or pentacene molecules in the simulated box. Each trajectory is recorded at 1 fs time steps, for 18 ps. With each MD trajectory (time-evolution of a box of equilibrated, neutral molecules), we collect more than 300 trajectories of Δ*E*_int_(*t*), by placing a positive charge on a different molecule. Molecules selected to carry positive charges were separated, as we required at least 10 Å for ethylene, or 7 Å for antracene and pentacene, between the molecules selected.

To properly take ensemble averages, we have repeated the accumulation of data from *multiple* independent trajectories of MD simulation, with 300 sampled Δ*E*_int_(*t*) from each trajectory. The uncertainty of λ_*out*_ values was calculated from the standard deviation (SD) of averaged *C*(0) in eq 18, until the standard deviation of the averaged λ_*out*_ is 1% or less of their mean values. In so doing, we accumulated more than 3,700 MD trajectories in each case and the final number of trajectories varied case by case. Under this setting, the averaged long-time correlation function values (*C*(*t*) at 9 ps) are of the order 10^–4^ eV^2^.

#### 2.2.2 Other Details of Molecular Dynamic Simulation

Force field parameters were generated with Q-Chem and Q-Force packages.^65,66^ In quantum chemistry calculations, ground state geometry optimization and vibrational analyses were performed with long-range corrected density functional LC-BLYP/DZ* with dispersion correction^67,68^ included. Long-range correction parameters, μ, employed for ethylene, anthracene, and pentacene are 0.41, 0.2, and 0.2 Bohr^–1^, respectively. Q-Force was employed to generate force-field parameters by fitting with force generated by the *ab initio* method.

In turning a neutral molecule to a cation, we calculate charges from the electrostatic potential on a grid (CHELPG),^69^ and add charge differences of its neutral ground state and its cationic state to the force field of the cation. In this calculation, we took the globally optimized structure calculated at LC-BLYP/DZ* with dispersion correction. In order to capture the system-bath interaction for λ_*out*_, we did not update the intramolecular force field parameters, such as equilibrium bond lengths and bond angles when a molecule is in the cationic state, as they contribute to λ_*in*_.

We employed OpenMM to simulate molecular dynamics.^70^ To reach an equilibrated state, the system was prepared with an Andersen thermostat and a Monte Carlo barostat, i.e., with an NPT ensemble at 1.0 bar for 10 ns. In this process, the ethylene system was equilibrated at 90 K, in an amorphous solid state. Temperatures for anthracene are 140 and 260 K, and for pentancene, 50 and 300 K, set by the following experimental works.^49,50^ With all systems equilibrated in their solid states, crystalline and amorphous phases are both considered for anthracene and pentacene. The crystalline structures start from the corresponding X-ray structures.^71,72^ Amorphous structures were initialized using PACKMOL software (version 20.3.1).^73,74^

## 3 Results

### 3.1 Reorganization Energy from Ultraviolet Photoelectron Spectroscopy

Ultraviolet photoelectron spectroscopy (UPS) has been employed to study the inner-sphere reorganization energy of anthracene and pentacene.^49,50^ With eqs 4 and 5, both low-frequency and high-frequency contributions of reorganization energy can be fitted from vibronic progression of UPS. Experimental spectra were fitted with a pseduo-Voigt function, which is a linear combination of Lorentzian and Gaussian functions, and fwhm values were reported. As shown in Table 1, λ_*s*_ values range from 177 to 549 meV. The result from gas phase pentacene spectra was also included, and a much smaller λ_*s*_, 18 meV, from a much narrower width of vibronic bands was also included in Table 1. A gas-phase system does not have any outer-shell reorganization and the bandwidth serves as a lower bound for this approach. The width and the inferred λ_*s*_ of solid-state UPS are well above this limit; therefore, inferring the broadening effect of the surrounding is not likely to be an instrumental artifact, and it should be reliable for inferring interactions with the environment. All λ_*s*_ values are of the same order of magnitude as λ_*v*_, implying the importance of low-frequency reorganization energy, which is mainly from outer-sphere interactions. The temperature dependence of λ_*s*_ is larger than that of λ_*v*_. Changing temperature could change the frequency of molecular vibrations, but its coupling to the charged state is not likely to change much. However, low-frequency modes contain intermolecular motions, which can be totally different at different temperatures; thus, their influence on the charged-state energy can be quite significant. In this case we observed a reduction in higher temperature cases.

**Table 1:** High- and Low-Frequency Contribution of Reorganization Energies Inferred from UPS Spectra^a^

system					
		fwhm	λ_*v*_	λ_*s*_	ref.
Anthracene	140 K/monolayer	184	150	505	(49)
Anthracene	260 K/monolayer	210	150	356	(49)
Pentacene	49 K/monolayer	113	109	549	(50)
Pentacene	298 K/monolayer	159	118	177	(50)
Pentacene	507 K/gas	65	97	18	(51)

a In units of meV.

### 3.2 Reorganization Energy from MD Simulation

#### 3.2.1 Ethylene at 90 K

**Figure 2:**
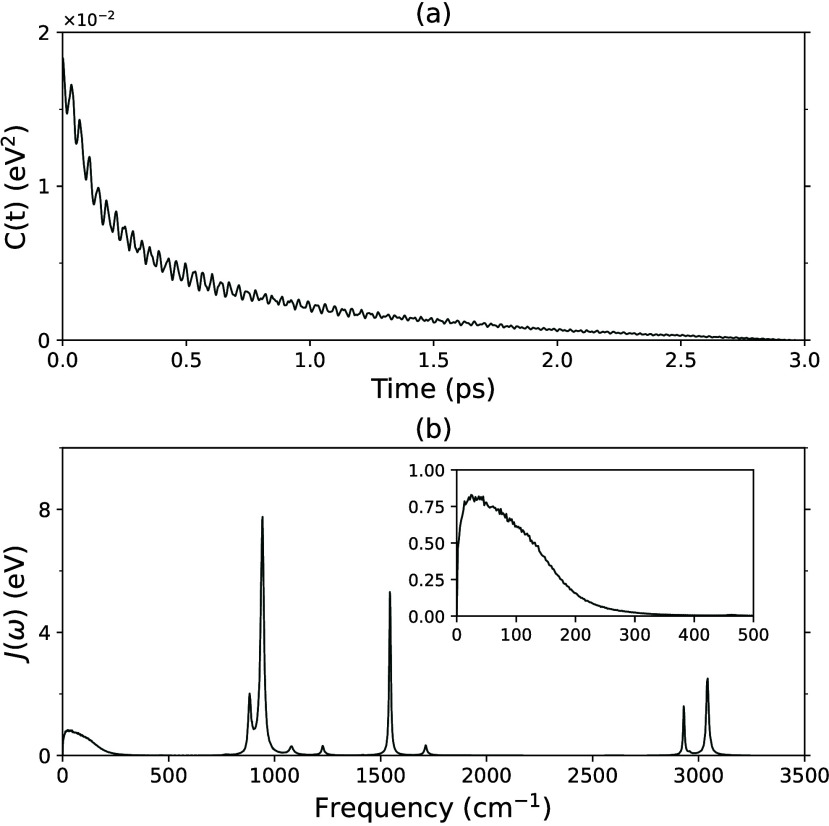
(a) The autocorrelation function of the interaction energy Δ*E*_int_, *C*(*t*), for amorphous ethylene at 90 K; (b) the corresponding spectral density *J*(ω). In the inset is a zoom-in for low frequency.

In Figure 2(a), the decay lifetime of ACF is fitted as 484 fs. As a comparison, the ACF for electronic coupling was reported in ref (75)., and the corresponding decay lifetimes are 1083 fs (55K) and 210 fs (155 K). In Figure 2(b), the spectral density *J*(ω) of amorphous ethylene at 90 K is shown. There exist low-frequency components (shown in the inset) together with several distinct peaks corresponding to their vibrational frequencies. λ_*out*_ values calculated with eq 17 are listed in Table 2. λ_*out*_ is obtained as 1088 meV. If eq 17 were integrated to 500 cm^–1^, the low-frequency contribution is 968 meV (86%). This result implies that intermolecular motions are the dominant source of reorganization.

**Table 2:** Outer-Shell Reorganization Energy from MD Simulations^a^

model frequency		crystalline		amorphous	
		all	low^b^	all	low
Ethylene	90 K			1088^c^	968
Anthracene	140 K	212	88	358	260
Anthracene	260 K	228	102	354	260
Pentacene	50 K	170	84	184	108
Pentacene	300 K	188	102	210	140

a Calculated with trajectories of 18 ps, with each trajectory generating more than 300 sampled cations.

b Contribution from frequencies under 500 cm^–1^ for ethylene or under 200 cm^–1^ for anthracene and pentacene.

c In units of meV.

#### 3.2.2 Anthracene at 140 and 260 K

**Figure 3:**
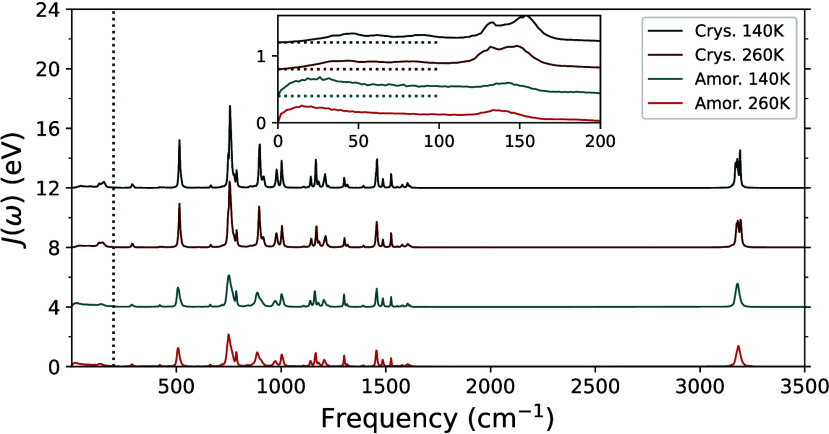
Spectral density functions of anthracene in crystalline (Crys.) and amorphous (Amor.) phases. Blue and orange lines denote 140 and 260 K, respectively. Dark lines are results for the crystalline state, and bright colors are for the amorphous phase. In the inset is a zoom-in for low frequency. Traces for the four data sets are shifted up for clarity. In the inset, their basal values are indicated by dashed lines of the same color to the left. The vertical dashed line indicates the cutoff frequency at 200 cm^–1^.

As seen in Figure 3, spectral densities are quite similar at different temperatures. Meanwhile, differences between amorphous and crystalline phases are more obvious, especially in the low-frequency region. The spectral density function is broader in the low-frequency region in the amorphous phase, compared to those in the crystalline phase.

In Table 2, λ_*out*_ calculated for crystalline and amorphous phases at 140 K are 212 and 358 meV, respectively, and low-frequency contributions are 88 (42%) and 260 meV (73%). Since spectral density functions are similar between 140 and 260 K, reorganization energies at 260 K are also similar as at 140 K, which are 228 and 354 meV for crystalline and amorphous phases, respectively, and low-frequency contributions are 102 (45%) and 260 meV (73%).

#### 3.2.3 Pentacene at 50 and 300 K

**Figure 4:**
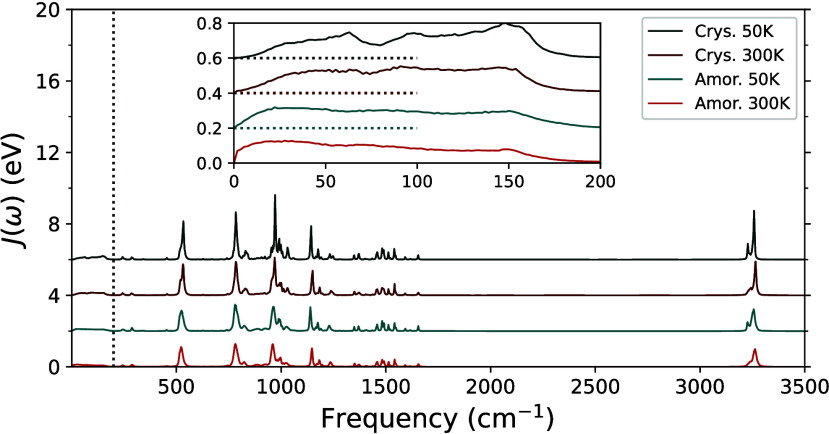
Spectral density functions of pentacene in crystalline (Crys.) and amorphous (Amor.) phases. Blue and orange lines are for 50 and 300 K, respectively. Dark lines are results for the crystalline phase, and bright colors are for the amorphous phase. In the inset is a zoom-in for low frequency. Traces for the four data sets are shifted up for clarity. In the inset, their basal values are indicated by dashed lines of the same color, to the left. The vertical dashed line indicates the cutoff frequency at 200 cm^–1^.

In Figure 4, spectral densities *J*(ω) of crystalline and amorphous pentacene at 50K are included, again showing the importance of low-frequency modes. Like that for anthracene, the low frequency spectral density is broader in the amorphous phase than in the crystalline phase.

In Table 2, the λ_*out*_ calculated for crystalline and amorphous phases at 50K are 170 and 184 meV, respectively, and the low-frequency contribution is 84 (49%) and 108 meV (59%). At 300 K, values of λ_*out*_ are 188 and 210 meV for crystalline and amorphous phases and the low-frequency contributions are 102 (54%) and 140 meV (67%).

## 4 Discussion

### 4.1 General Values of λ_*out*_ and λ_*s*_

**Figure 5:**
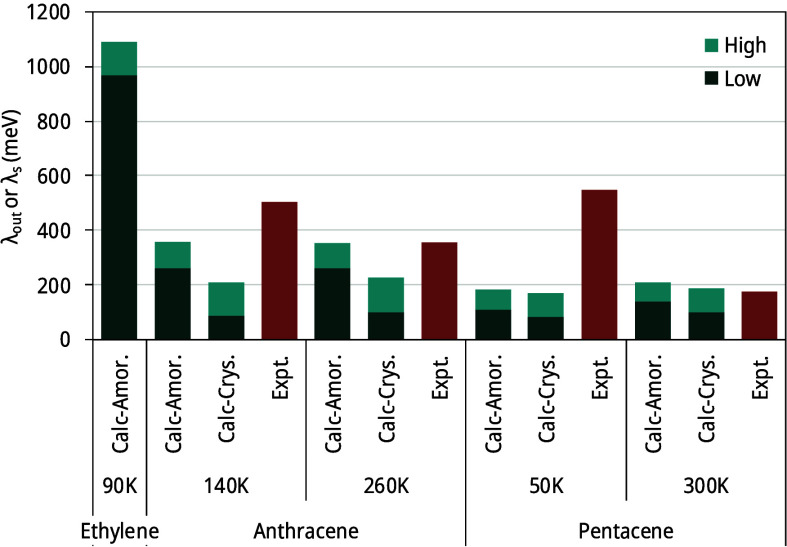
Experimental (Expt.) and calculated (Calc.) reorganization energies for both crystalline (Crys.) and amorphous (Amor.) states at the temperature indicated. λ_*out*_ values are reported for calculated data, while for experimental data, inferred λ_*s*_ values are included. Dark and light green colors indicate contributions to λ_*out*_ from low- and high-frequency portions in spectral density, respectively.

Our results for λ_*s*_ and λ_*out*_ are summarized in Figure 5. Values of λ_*s*_, treated as an upper bound for λ_*out*_, are indeed higher than λ_*out*_ calculated (the stacked height of the green bars) except for 300 K pentacene, where the two values are very close. We note that values of λ_*s*_ derived from experimental spectra are from the crystalline phase.

The largest value of λ_*out*_, 1,088 meV, was for amorphous ethylene, the smallest molecule in the present work. Calculated λ_*out*_ values for anthracene are all larger than that of pentacene. A λ_*out*_ value that is higher than 1 eV maybe unusual. As the smallest model molecule with a π bond, such a value does not have any experimental implications either. Nevertheless, values of λ_*out*_, and λ_*s*_ are all higher than 100 meV, which is about 4 times room temperature already, and they are certainly not negligible in practice.

### 4.2 High-Frequency Contribution of λ_*out*_ in Nonpolar Systems

A high-frequency contribution to λ_*out*_ has rarely been discussed in the literature, to the best of our knowledge. In traditional polar solvation works, vibrational peaks in infra absorption can be transformed into the dielectric function ε(ω) and peaks are seen in the spectral density function calculated from simple solvation models.^62,63^ However, in these cases, high-frequency contributions to the overall λ_*out*_ are quite small, such that their quantum statistics do not really affect spectra of dynamics Stokes shift.^62^ For nonpolar systems, the same dielectric function yields very small λ_*out*_ with narrow vibration peaks seen in the *J*(ω) modeled as well.^61^ Therefore, in the present work, λ_*out*_ in the nonpolar systems simulated has a significant high-frequency component, as seen in Figure 5 and Table 2. This is a rather unexpected observation. High-frequency components of λ_*out*_ are similar, or even larger than low-frequency components in some cases, especially for the crystal phase (>50% for both temperatures in anthracene, and in pantenecene, 51% for 50K, and 46% for 300 K data.).

High-frequency components of λ_*out*_ contribute to the third term on the right-handed side of Eq 8. The second term in Eq 8 can be estimated through density functional theory (DFT) for λ_*in*_ with vibrational analysis,^76^ and the values reported for both anthracene and pantacene are pretty small (0 or 1 meV for each mode, with 2 modes under 500 cm^–1^) in gas phase calculation, leading to a conclusion that real values of λ_*s*_ can be much smaller than we have obtained from fitting UPS. However, MD and quantum chemistry are quite different in their model assumptions and sources of errors. A direct comparison and subtraction may not yield physically reliable values and further detailed studies may be necessary for a proper conclusion. Here we aim to obtain λ_*out*_ and it is sufficient to conclude their significant magnitude.

### 4.3 Low Frequency Behavior of *J*(ω)

Environmental fluctuation to a system is often characterized using the Ohmic model for the spectral density.^77^
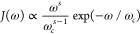
19where ω_*c*_ is a characteristic “cut-off” frequency, and *s* indicates the power-law dependence at low frequencies. The Ohmic spectral density is commonly employed in many theoretical works on quantum dynamics of many systems.^78−80^ Reporting fitted models from simulation offers practical model settings in these studies and better predictions and insights can be drawn. We fit our calculated spectral density function to Eq 19 and results are listed in Table 3.

**Table 3:** Descriptive Parameters of the Ohmic Model for the Spectral Density Function

model	temp.	phase	*s*	ω_*c*_ (cm^–1^)
Anthracene	140 K	crystalline	2.032	28.6
		amorphous	0.452	55.6
	260 K	crystalline	1.582	35.7
		amorphous	0.259	66.4
Pentacene	50 K	crystalline	2.655	19.4
		amorphous	0.788	48.1
	300 K	crystalline	1.600	33.5
		amorphous	0.325	83.1

As seen in Table 3, we found that all *s* values for the crystalline phase are larger than 1, indicating a super-Ohmic case, whereas *s* for amorphous systems are smaller than 1, indicating a sub-Ohmic situation. As discussed earlier, in Figures 3 and 4, different behaviors in *J*(ω) in low-frequency limits are seen. The narrower low-frequency bands for *J*(ω) in crystallines are mainly in the left side of the band, where the slope in *J*(ω) goes from flat to steep before reaching the peak, showing a higher-order dependence of ω.

In ref (75), we reported the spectral density function for the fluctuation of electronic coupling, or the off-diagonal Hamiltonian matrix element. There we reported sub-Ohmic dynamics for amorphous phases. Our present findings of both sub- and super-Ohmic dynamics for fluctuations of system-bath interactions in different phases can be useful for future studies on dynamics in ET systems. They also motivate us to reconsider sub-Ohmic dynamics we obtained for the off-diagonal Hamiltionian. It would be interesting to study fluctuation dynamics in electron transfer in a crystalline phase in the future.

**Figure 6:**
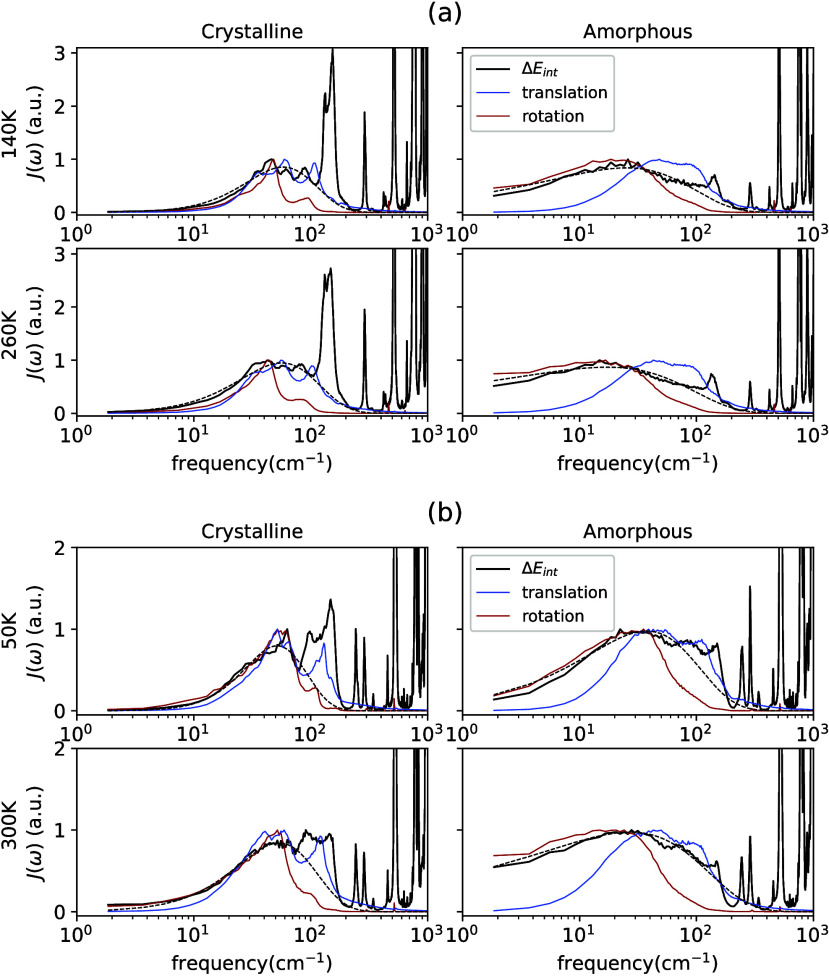
Spectral density curves of energetic disorder (black thick lines), their ohmic fitted curves (dashed lines), and those derived from translation (blue lines) and rotation (red lines) for (a) anthracene and (b) pentacene. In both (a) and (b), the upper and lower panels represent low and high temperatures, respectively, and the left and right columns depict crystalline and amorphous phases, respectively.

In amorphous solid systems we can see that our MD simulated *J*(ω) does not approach zero at low frequency. In Figure 6, the lowest frequency data point for *J*(ω) is located at about 1.8 cm^–1^ and values of spectral densities from the correlation function of Δ*E*_*int*_, as well as those from rotational correlation, remain at a finite value in the amorphous cases. This is due to a long-time correlation from MD trajectories, especially derived from amorphous phases. This result indicates that the entire correlation of such movement is not fully captured and an ensemble of even longer trajectories is needed. However, for the purpose of the present work, in estimating λ_*out*_ and further analyzing the physical origin, our present computational setting is sufficient. It would be interesting to further study such long-time correlation behavior in the future, since it allows us to further study properties related to inhomogeneous broadening, a set of important characteristics in amorphous solid states of material, but hard to study in detail.

### 4.4 Possible Sources of λ_*out*_

Our resulting λ_*out*_ values are similar to those estimated for nonpolar solvents.^34,35^ The MD force field contains source physics of λ_*out*_. Since we are employing a classical force field, the effect of quadrupolar solvation is included.^35^ For the indirect effect of induced polarization changes with structure factor,^34^ a polarizable force field would need to be employed, but it is not present in our current simulation, since Δ*E*_*int*_ is simulated with only electrostatic interactions of the cation. Therefore, we believe that the observed λ_*out*_ is from the quadrupolar or other higher-order moments.

Since the MD simulation was performed with the goal of determining λ_*out*_, arising from the electrostatic interaction between the cation and surrounding molecules, we did not include changes in structure when turning a neutral molecule into a cation, as they contribute to the inner-sphere reorganization energy. In so doing, we have ignored possible contributions to λ_*out*_ from the movement of surrounding molecules in response to a change in molecular geometry of the cation. Without this contribution, albeit minor, λ_*out*_ values we have obtained are not negligible already.

In addition, the previous study revealed the importance of rotational and translation motions in the low-frequency band of the spectral density of off-diagonal disorder.^75^ Therefore, dynamics, their corresponding ACF, and spectral densities were also investigated in the present work. To capture translational characteristics, the ACF is defined as
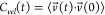
20where v⃗ is the velocity of central mass for each molecule.

The rotational ACF, *C*_*rot*_(*t*), is defined through dynamics of the principle axis with the smallest moment of inertia, which is also aligned with the long molecular axis, as
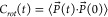
21With P⃗ being a normalized principle axis vector in the molecule. *C*_*rot*_(*t*) is an averaged cosine function of the angle between the two vectors.

Figure 6 shows rotational and translation spectral densities, together with the spectral densities of Δ*E*_*int*_, and the fitted Ohmic function. The spectral density for Δ*E*_*int*_ is a general combination of the two, with its higher frequency edge near 100 cm^–1^ following that of the translation spectral profile, and the lower frequency rotational. In the crystalline phase, all three curves are very similar in the region of ∼1–30 cm^–1^. In this region, slopes of these curves become steeper as frequency increases, indicating a clear super-Ohmic character.

It is somewhat surprising to see that rotational spectra in the amporphous phase have a significant contribution in a lower frequency region, as compared to translational spectra (1–10 cm^–1^), in all four cases studied. Very similar behavior for Δ*E*_*int*_ is also seen in this region. This is consistent with data shown in Figure 5, where values of λ_*out*_ are larger in the amorphous phase than the crystalline phase, mainly in their low-frequency contribution. Thus, it is likely that additional, low-frequency components in λ_*out*_ are mainly constrained rotation. In an amorphous solid, without the ordered planar stacking in crystallines, molecules may have more space where hindered rotation is allowed. In this region, curves do not increase significantly before they reach the broad peak, indicating a sub-Ohmic characteristics.

### 4.5 Effects of λ_*out*_ in Estimating ET Rates

**Figure 7:**
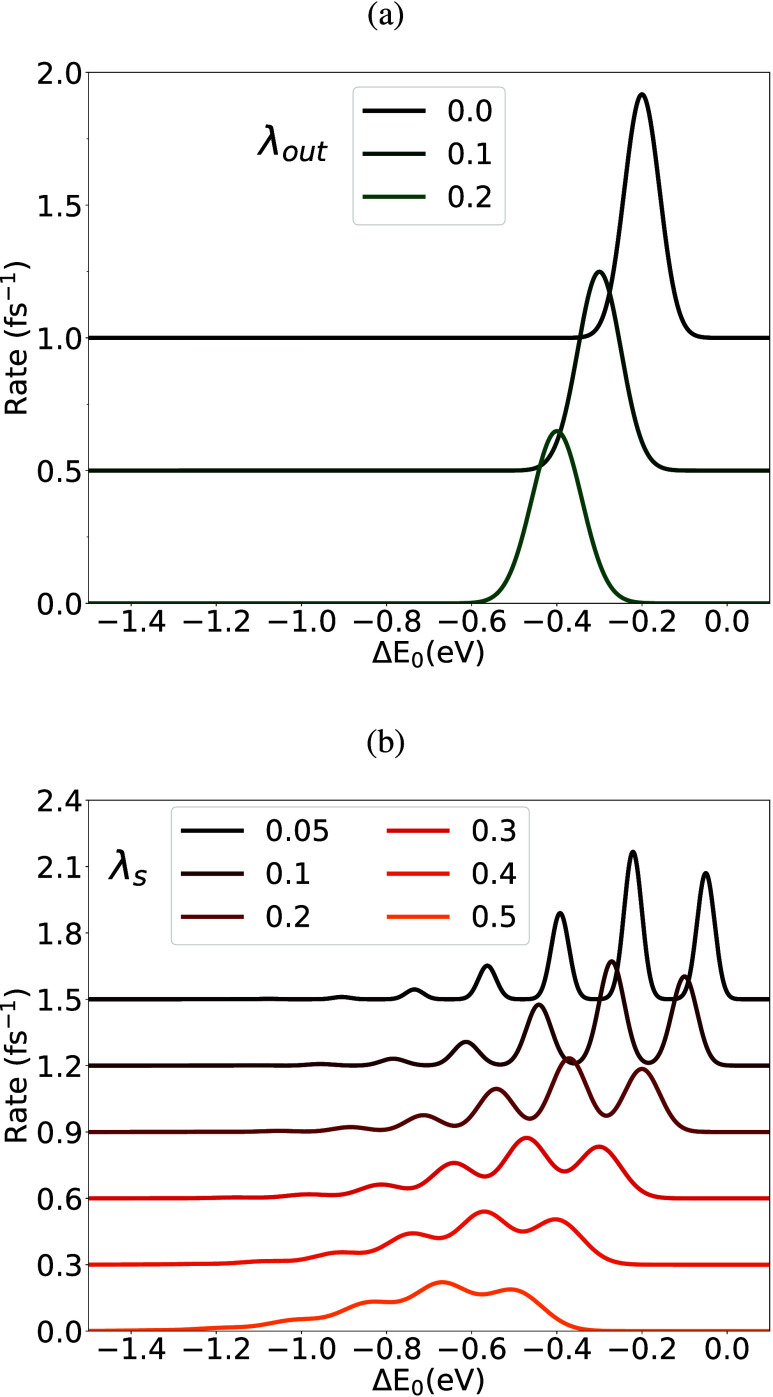
(a) ET rates with different λ_*out*_ values (in eV) for the classical Marcus ET rate, eq 1, with a fixed λ_*in*_ as 0.2 eV. (b) ET rates from the Jortner’s expression, eq 3, with different λ_*s*_ (in eV) as shown. λ_*v*_ was set to 0.2 eV or with *S* being 1.169, and *ℏω*_ν_ was set to 1380 cm^–1^. Other parameters employed are *V*, 0.1 eV, and *T*, 50 K.

ET rates with both the Marcus and Jortner’s expressions (eqs 1 and 3) under different λ_*out*_ (or λ_*s*_), as a function of Δ*E*_0_ are shown in Figure 7. λ_*out*_ not only shifts the overall profile to a more negative Δ*E*_0_ region, it also broadens the Gaussian profile for the Marcus rate. λ_*s*_ has a similar effect for the Jortner’s rate: the vibronic progression bands are moved toward a more negative Δ*E*_0_ as they become broader. With zero λ_*out*_, it is not a problem to obtain a Marcus rate, but zero λ_*out*_ implies a small λ_*s*_, and in the Jortner’s expression, it would lead a sum of very narrow Gaussian functions. The steep dependence on Δ*E*_0_ is not physically plausible, and it becomes hard to ensure predicted quality. A properly determined λ_*out*_ (which is included in λ_*s*_) is essential to predict ET rate, even for nonpolar systems.

### 4.6 Microscopic Dielectric Solvation

Our observation of significant outer-sphere organization energy in nonpolar systems is a deviation from bulk dielectric solvation models. A plausible way to understand this result is that, compared to the bulk, in a microscopic system, a larger dielectric solvation effect exists. This is consistent with many discussions for screening of charges in molecular dynamics, especially for biomolecules. For example, by studying the effect of polarizable solvation, dielectric constants that are *larger* than that from electronic polarization were reported.^81^ The dielectric constant formulation is related to the mean square of total dipole in a sampled volume, which contains the mean dipole, as well as the fluctuation of the dipole.

## 5 Conclusions

We studied outer-shell reorganization energies λ_*out*_ in nonpolar molecular charge transporting systems and found that values can approach or even exceed 100 meV. Furthermore, the contribution at low-frequency is larger than 50%. This result indicates that translational and rotational modes of surrounding molecules are important in accommodating the charged molecule. Both crystalline and amorphous structures were also included in the simulation, and results show only a minor difference in the value of λ_*out*_. However, further analysis of their spectral density functions revealed sub-Ohmic dynamics for amorphous phase and super-Ohmic for crystalline phase. Nevertheless, this work demonstrates that λ_*out*_ cannot be ignored, even in nonpolar systems.

## References

[ref1] Marcus R. A. On the Theory of Oxidation-Reduction Reactions Involving Electron Transfer. I. J. Chem. Phys. 1956, 24, 966–978. 10.1063/1.1742723.

[ref2] Sirringhaus H. Device Physics of Solution-Processed Organic Field-Effect Transistors. Adv. Mater. 2005, 17, 2411–2425. 10.1002/adma.200501152.

[ref3] Coropceanu V.; Cornil J.; Da Silva Filho D. A.; Olivier Y.; Silbey R.; Brédas J.-L. Charge Transport in Organic Semiconductors. Chem. Rev. 2007, 107, 926–952. 10.1021/cr050140x. 17378615

[ref4] Walzer K.; Maennig B.; Pfeiffer M.; Leo K. Highly Efficient Organic Devices Based on Electrically Doped Transport Layers. Chem. Rev. 2007, 107, 1233–1271. 10.1021/cr050156n. 17385929

[ref5] Li G.; Zhu R.; Yang Y. Polymer Solar Cells. Nat. Photonics 2012, 6, 153–161. 10.1038/nphoton.2012.11.

[ref6] Yoshida K.; Gong J.; Kanibolotsky A. L.; Skabara P. J.; Turnbull G. A.; Samuel I. D. W. Electrically Driven Organic Laser Using Integrated OLED Pumping. Nature 2023, 621, 746–752. 10.1038/s41586-023-06488-5. 37758890 PMC10533406

[ref7] Zhong D.; Wu C.; Jiang Y.; Yuan Y.; Kim M.-g.; Nishio Y.; Shih C.-C.; Wang W.; Lai J.-C.; Ji X.; Gao T. Z.; Wang Y.-X.; Xu C.; Zheng Y.; Yu Z.; Gong H.; Matsuhisa N.; Zhao C.; Lei Y.; Liu D.; Zhang S.; Ochiai Y.; Liu S.; Wei S.; Tok J. B.-H.; Bao Z. High-Speed and Large-Scale Intrinsically Stretchable Integrated Circuits. Nature 2024, 627, 313–320. 10.1038/s41586-024-07096-7. 38480964

[ref8] Chen H.; Liu C.; Xu J.; Maxwell A.; Zhou W.; Yang Y.; Zhou Q.; Bati A. S. R.; Wan H.; Wang Z.; Zeng L.; Wang J.; Serles P.; Liu Y.; Teale S.; Liu Y.; Saidaminov M. I.; Li M.; Rolston N.; Hoogland S.; Filleter T.; Kanatzidis M. G.; Chen B.; Ning Z.; Sargent E. H. Improved Charge Extraction in Inverted Perovskite Solar Cells with Dual-Site-Binding Ligands. Science 2024, 384, 189–193. 10.1126/science.adm9474. 38603485

[ref9] Forrest S. R. The Path to Ubiquitous and Low-Cost Organic Electronic Appliances on Plastic. Nature 2004, 428, 911–918. 10.1038/nature02498. 15118718

[ref10] Fuchs A.; Steinbrecher T.; Mommer M. S.; Nagata Y.; Elstner M.; Lennartz C. Molecular Origin of Differences in Hole and Electron Mobility in Amorphous Alq3—a Multiscale Simulation Study. Phys. Chem. Chem. Phys. 2012, 14, 4259–4270. 10.1039/c2cp23489k. 22337316

[ref11] Martinelli N. G.; Savini M.; Muccioli L.; Olivier Y.; Castet F.; Zannoni C.; Beljonne D.; Cornil J. Modeling Polymer Dielectric/Pentacene Interfaces: On the Role of Electrostatic Energy Disorder on Charge Carrier Mobility. Adv. Funct. Mater. 2009, 19, 3254–3261. 10.1002/adfm.200901077.

[ref12] Rühle V.; Lukyanov A.; May F.; Schrader M.; Vehoff T.; Kirkpatrick J.; Baumeier B.; Andrienko D. Microscopic Simulations of Charge Transport in Disordered Organic Semiconductors. J. Chem. Theory Comput. 2011, 7, 3335–3345. 10.1021/ct200388s. 22076120 PMC3210523

[ref13] Moral M.; Pérez-Jiménez A. J.; Sancho-García J. C. Understanding and Controlling Chemical Modifications of Rubicene for Their Envisioned Use as Molecular Organic Semiconductors. J. Phys. Chem. C 2017, 121, 3171–3181. 10.1021/acs.jpcc.6b10566.

[ref14] Gali S. M.; Matta M.; Lessard B. H.; Castet F.; Muccioli L. Ambipolarity and Dimensionality of Charge Transport in Crystalline Group 14 Phthalocyanines: A Computational Study. J. Phys. Chem. C 2018, 122, 2554–2563. 10.1021/acs.jpcc.7b11588.

[ref15] Stangel C.; Schubert C.; Kuhri S.; Rotas G.; Margraf J. T.; Regulska E.; Clark T.; Torres T.; Tagmatarchis N.; Coutsolelos A. G.; Guldi D. M. Tuning the Reorganization Energy of Electron Transfer in Supramolecular Ensembles – Metalloporphyrin, Oligophenylenevinylenes, and Fullerene – and the Impact on Electron Transfer Kinetics. Nanoscale 2015, 7, 2597–2608. 10.1039/C4NR05165C. 25581327

[ref16] Cerdá J.; Calbo J.; Ortí E.; Aragó J. Charge-Separation and Charge-Recombination Rate Constants in a Donor–Acceptor Buckybowl-Based Supramolecular Complex: Multistate and Solvent Effects. J. Phys. Chem. A 2021, 125, 9982–9994. 10.1021/acs.jpca.1c05740. 34767714 PMC8630798

[ref17] Wang C.; Wu B.; Wang C. Rational Construction and Efficient Regulation of Stable and Long-Lived Charge-Separation State in Fullerene Materials. Acc. Mater. Res. 2024, 5, 426–437. 10.1021/accountsmr.3c00247.

[ref18] Lin B. C.; Cheng C. P.; Lao Z. P. M. Reorganization Energies in the Transports of Holes and Electrons in Organic Amines in Organic Electroluminescence Studied by Density Functional Theory. J. Phys. Chem. A 2003, 107, 5241–5251. 10.1021/jp0304529.

[ref19] Gruhn N. E.; da Silva Filho D. A.; Bill T. G.; Malagoli M.; Coropceanu V.; Kahn A.; Brédas J.-L. The Vibrational Reorganization Energy in Pentacene: Molecular Influences on Charge Transport. J. Am. Chem. Soc. 2002, 124, 7918–7919. 10.1021/ja0175892. 12095333

[ref20] Marcus R. A. Electrostatic Free Energy and Other Properties of States Having Nonequilibrium Polarization. I. J. Chem. Phys. 1956, 24, 979–989. 10.1063/1.1742724.

[ref21] Alkan M.; Yavuz I. Intrinsic Charge-Mobility in Benzothieno[3,2-b][1]Benzothiophene (BTBT) Organic Semiconductors Is Enhanced with Long Alkyl Side-Chains. Phys. Chem. Chem. Phys. 2018, 20, 15970–15979. 10.1039/C8CP01640B. 29850708

[ref22] Oberhofer H.; Blumberger J. Revisiting Electronic Couplings and Incoherent Hopping Models for Electron Transport in Crystalline C60 at Ambient Temperatures. Phys. Chem. Chem. Phys. 2012, 14, 13846–13852. 10.1039/c2cp41348e. 22858858

[ref23] Malagoli M.; Coropceanu V.; Da Silva Filho D. A.; Brédas J. L. A Multimode Analysis of the Gas-Phase Photoelectron Spectra in Oligoacenes. J. Chem. Phys. 2004, 120, 7490–7496. 10.1063/1.1687675. 15267661

[ref24] Chang Y.-C.; Chao I. An Important Key to Design Molecules with Small Internal Reorganization Energy: Strong Nonbonding Character in Frontier Orbitals. J. Phys. Chem. Lett. 2010, 1, 116–121. 10.1021/jz900042x.

[ref25] Chen K.; Kunkel C.; Reuter K.; Margraf J. T. Reorganization Energies of Flexible Organic Molecules as a Challenging Target for Machine Learning Enhanced Virtual Screening. Digital Discovery 2022, 1, 147–157. 10.1039/D1DD00038A.

[ref26] Staker J.; Marshall K.; Leswing K.; Robertson T.; Halls M. D.; Goldberg A.; Morisato T.; Maeshima H.; Ando T.; Arai H.; Sasago M.; Fujii E.; Matsuzawa N. N. *De Novo* Design of Molecules with Low Hole Reorganization Energy Based on a Quarter-Million Molecule DFT Screen: Part 2. J. Phys. Chem. A 2022, 126, 5837–5852. 10.1021/acs.jpca.2c04221. 35984470

[ref27] Holroyd R. A.; Miller J. R. Rate versus Free Energy Change for Attaching Highly Mobile Electrons to Molecules in Nonpolar Liquids. J. Phys. Chem. B 2019, 123, 9206–9211. 10.1021/acs.jpcb.9b07845. 31594307

[ref28] Reynolds L.; Gardecki J. A.; Frankland S. J. V.; Horng M. L.; Maroncelli M. Dipole Solvation in Nondipolar Solvents: Experimental Studies of Reorganization Energies and Solvation Dynamics. J. Phys. Chem. 1996, 100, 10337–10354. 10.1021/jp953110e.

[ref29] Imahori H.; Tkachenko N. V.; Vehmanen V.; Tamaki K.; Lemmetyinen H.; Sakata Y.; Fukuzumi S. An Extremely Small Reorganization Energy of Electron Transfer in Porphyrin-Fullerene Dyad. J. Phys. Chem. A 2001, 105, 1750–1756. 10.1021/jp003207n.

[ref30] Benduhn J.; Tvingstedt K.; Piersimoni F.; Ullbrich S.; Fan Y.; Tropiano M.; McGarry K. A.; Zeika O.; Riede M. K.; Douglas C. J.; Barlow S.; Marder S. R.; Neher D.; Spoltore D.; Vandewal K. Intrinsic Non-Radiative Voltage Losses in Fullerene-Based Organic Solar Cells. Nat. Energy 2017, 2, 17053 10.1038/nenergy.2017.53.

[ref31] Matyushov D. V. Dipole Solvation in Dielectrics. J. Chem. Phys. 2004, 120, 1375–1382. 10.1063/1.1633545. 15268263

[ref32] Matyushov D. V. Solvent Reorganization Energy of Electron-Transfer Reactions in Polar Solvents. J. Chem. Phys. 2004, 120, 7532–7556. 10.1063/1.1676122. 15267667

[ref33] Lee M. H.; Dunietz B. D.; Geva E. Calculation from First Principles of Intramolecular Golden-Rule Rate Constants for Photo-Induced Electron Transfer in Molecular Donor–Acceptor Systems. J. Phys. Chem. C 2013, 117, 23391–23401. 10.1021/jp4081417.

[ref34] Matyushov D. V. Electron Transfer in Nonpolar Media. Phys. Chem. Chem. Phys. 2020, 22, 10653–10665. 10.1039/C9CP06166E. 31934688

[ref35] Milischuk A. A.; Matyushov D. V. Equilibrium Solvation in Quadrupolar Solvents. J. Chem. Phys. 2005, 123, 044501 10.1063/1.1961442. 16095363

[ref36] Leontyev I. V.; Tachiya M. The Reorganization Energy of Electron Transfer in Nonpolar Solvents: Molecular Level Treatment of the Solvent. J. Chem. Phys. 2005, 123, 224502 10.1063/1.2131054. 16375484

[ref37] Leontyev I. V.; Tachiya M. Molecular Level Approaches for Investigation of Electron Transfer in Nonpolar Solvents. J. Chem. Phys. 2007, 126, 064501 10.1063/1.2423026. 17313223

[ref38] Ahmed R.; Manna A. K. Origins of Large Stokes Shifts in a Pyrene–Styrene-Based Push–Pull Organic Molecular Dyad in Polar Solvents and Large Electron Mobility in the Crystalline State: A Theoretical Perspective. J. Phys. Chem. C 2022, 126, 423–433. 10.1021/acs.jpcc.1c09288.

[ref39] Baggioli A.; Casalegno M.; Raos G.; Muccioli L.; Orlandi S.; Zannoni C. Atomistic Simulation of Phase Transitions and Charge Mobility for the Organic Semiconductor Ph-BTBT-C10. Chem. Mater. 2019, 31, 7092–7103. 10.1021/acs.chemmater.9b02882.

[ref40] Guo Y.; Wang W.; Shao R.; Yin S. Theoretical Study on the Electron Transport Properties of Chlorinated Pentacene Derivatives. Comput. Theor. Chem. 2015, 1057, 67–73. 10.1016/j.comptc.2015.01.019.

[ref41] Huang W.; Xie W.; Huang H.; Zhang H.; Liu H. Designing Organic Semiconductors with Ultrasmall Reorganization Energies: Insights from Molecular Symmetry, Aromaticity and Energy Gap. J. Phys. Chem. Lett. 2020, 11, 4548–4553. 10.1021/acs.jpclett.0c01199. 32437617

[ref42] Kordt P.; Van Der Holst J. J. M.; Al Helwi M.; Kowalsky W.; May F.; Badinski A.; Lennartz C.; Andrienko D. Modeling of Organic Light Emitting Diodes: From Molecular to Device Properties. Adv. Funct. Mater. 2015, 25, 1955–1971. 10.1002/adfm.201403004.

[ref43] Landi A.; Borrelli R.; Capobianco A.; Velardo A.; Peluso A. Hole Hopping Rates in Organic Semiconductors: A Second-Order Cumulant Approach. J. Chem. Theory Comput. 2018, 14, 1594–1601. 10.1021/acs.jctc.7b00858. 29345937

[ref44] MacKenzie R. C. I.; Frost J. M.; Nelson J. A Numerical Study of Mobility in Thin Films of Fullerene Derivatives. J. Chem. Phys. 2010, 132, 064904 10.1063/1.3315872. 20151755

[ref45] Sun F.; Jin R. Optical and Charge Transport Properties of N-butyl-1,8-Naphthalimide Derivatives as Organic Light-Emitting Materials: A Theoretical Study. J. Lumin. 2014, 149, 125–132. 10.1016/j.jlumin.2014.01.011.

[ref46] Tang X.-D.; Liao Y.; Geng H.; Shuai Z.-G. Fascinating Effect of Dehydrogenation on the Transport Properties of N-heteropentacenes: Transformation from p- to n-Type Semiconductor. J. Mater. Chem. 2012, 22, 18181 10.1039/c2jm33039c.

[ref47] McMahon D. P.; Troisi A. Evaluation of the External Reorganization Energy of Polyacenes. J. Phys. Chem. Lett. 2010, 1, 941–946. 10.1021/jz1001049.

[ref48] Norton J. E.; Brédas J.-L. Polarization Energies in Oligoacene Semiconductor Crystals. J. Am. Chem. Soc. 2008, 130, 12377–12384. 10.1021/ja8017797. 18715006

[ref49] Bussolotti F.; Han S. W.; Honda Y.; Friedlein R. Phase-Dependent Electronic Properties of Monolayer and Multilayer Anthracene Films on Graphite [0001] Surfaces. Phys. Rev. B 2009, 79, 245410 10.1103/PhysRevB.79.245410.

[ref50] Yamane H.; Nagamatsu S.; Fukagawa H.; Kera S.; Friedlein R.; Okudaira K. K.; Ueno N. Hole-Vibration Coupling of the Highest Occupied State in Pentacene Thin Films. Phys. Rev. B 2005, 72, 153412 10.1103/PhysRevB.72.153412.

[ref51] Kera S.; Yamane H.; Ueno N. First-Principles Measurements of Charge Mobility in Organic Semiconductors: Valence Hole–Vibration Coupling in Organic Ultrathin Films. Prog. Surf. Sci. 2009, 84, 135–154. 10.1016/j.progsurf.2009.03.002.

[ref52] Jortner J. Temperature Dependent Activation Energy for Electron Transfer between Biological Molecules. J. Chem. Phys. 1976, 64, 4860–4867. 10.1063/1.432142.

[ref53] Marcus R. A. The Second R. A. Robinson Memorial Lecture. Electron, Proton and Related Transfers. Faraday Discuss. Chem. Soc. 1982, 74, 7–15. 10.1039/dc9827400007.

[ref54] Barbara P. F.; Meyer T. J.; Ratner M. A. Contemporary Issues in Electron Transfer Research. J. Phys. Chem. 1996, 100, 13148–13168. 10.1021/jp9605663.

[ref55] Ulstrup J.; Jortner J. The Effect of Intramolecular Quantum Modes on Free Energy Relationships for Electron Transfer Reactions. J. Chem. Phys. 1975, 63, 4358–4368. 10.1063/1.431152.

[ref56] Stehr V.; Fink R. F.; Tafipolski M.; Deibel C.; Engels B. Comparison of Different Rate Constant Expressions for the Prediction of Charge and Energy Transport in Oligoacenes. Wiley Interdiscip. Rev. Comput. Mol. Sci. 2016, 6, 694–720. 10.1002/wcms.1273.

[ref57] Lee E.; Medvedev E. S.; Stuchebrukhov A. A. Effect of Quantum Modes in Biological Electron Transfer Reactions: A Useful Approximation for the Harmonic Model with Frequency Change and Duchinsky Rotation. J. Chem. Phys. 2000, 112, 9015–9024. 10.1063/1.481513.

[ref58] Hsu C.-P.; Yang C.-H. Exploring Chemical Concepts Through Theory and Computation; John Wiley & Sons, Inc., 2024; pp 317–334.

[ref59] Huang K.; Rhys A. Theory of Light Absorption and Non-Radiative Transitions in F-Centres. Proc. R. Soc. London, Ser. A 1950, 204, 406–423. 10.1098/rspa.1950.0184.

[ref60] Coropceanu V.; Malagoli M.; Da Silva Filho D. A.; Gruhn N. E.; Bill T. G.; Brédas J. L. Hole- and Electron-Vibrational Couplings in Oligoacene Crystals: Intramolecular Contributions. Phys. Rev. Lett. 2002, 89, 275503 10.1103/PhysRevLett.89.275503. 12513217

[ref61] Hsu C.-P. Reorganization Energies and Spectral Densities for Electron Transfer Problems in Charge Transport Materials. Phys. Chem. Chem. Phys. 2020, 22, 21630 10.1039/D0CP02994G. 32969457

[ref62] Hsu C.-P.; Georgievskii Y.; Marcus R. A. Time-Dependent Fluorescence Spectra of Large Molecules in Polar Solvents. J. Phys. Chem. A 1998, 102, 2658–2666. 10.1021/jp980255n.

[ref63] Hsu C.-P.; Song X.; Marcus R. A. Time-Dependent Stokes Shift and Its Calculation from Solvent Dielectric Dispersion Data. J. Phys. Chem. B 1997, 101, 2546–2551. 10.1021/jp9630885.

[ref64] Ovchinnikov A. A.; Ovchinnikova M. Y. Contribution to the Theory of Elementary Electron Transfer Reactions in Polar Liquids. Solv. Phys. JEPT 1969, 29 (4), 688–693.

[ref65] Sami S.; Menger M. F.; Faraji S.; Broer R.; Havenith R. W. A. Q-Force: Quantum Mechanically Augmented Molecular Force Fields. J. Chem. Theory Comput. 2021, 17, 4946–4960. 10.1021/acs.jctc.1c00195. 34251194 PMC8359013

[ref66] Epifanovsky E.; Gilbert A. T. B.; Feng X.; Lee J.; Mao Y.; Mardirossian N.; Pokhilko P.; White A. F.; Coons M. P.; Dempwolff A. L.; Gan Z.; Hait D.; Horn P. R.; Jacobson L. D.; Kaliman I.; Kussmann J.; Lange A. W.; Lao K. U.; Levine D. S.; Liu J.; McKenzie S. C.; Morrison A. F.; Nanda K. D.; Plasser F.; Rehn D. R.; Vidal M. L.; You Z.-Q.; Zhu Y.; Alam B.; Albrecht B. J.; Aldossary A.; Alguire E.; Andersen J. H.; Athavale V.; Barton D.; Begam K.; Behn A.; Bellonzi N.; Bernard Y. A.; Berquist E. J.; Burton H. G. A.; Carreras A.; Carter-Fenk K.; Chakraborty R.; Chien A. D.; Closser K. D.; Cofer-Shabica V.; Dasgupta S.; de Wergifosse M.; Deng J.; Diedenhofen M.; Do H.; Ehlert S.; Fang P.-T.; Fatehi S.; Feng Q.; Friedhoff T.; Gayvert J.; Ge Q.; Gidofalvi G.; Goldey M.; Gomes J.; González-Espinoza C. E.; Gulania S.; Gunina A. O.; Hanson-Heine M. W. D.; Harbach P. H. P.; Hauser A.; Herbst M. F.; Hernández Vera M.; Hodecker M.; Holden Z. C.; Houck S.; Huang X.; Hui K.; Huynh B. C.; Ivanov M.; Jász Á.; Ji H.; Jiang H.; Kaduk B.; Kähler S.; Khistyaev K.; Kim J.; Kis G.; Klunzinger P.; Koczor-Benda Z.; Koh J. H.; Kosenkov D.; Koulias L.; Kowalczyk T.; Krauter C. M.; Kue K.; Kunitsa A.; Kus T.; Ladjánszki I.; Landau A.; Lawler K. V.; Lefrancois D.; Lehtola S.; Li R. R.; Li Y.-P.; Liang J.; Liebenthal M.; Lin H.-H.; Lin Y.-S.; Liu F.; Liu K.-Y.; Loipersberger M.; Luenser A.; Manjanath A.; Manohar P.; Mansoor E.; Manzer S. F.; Mao S.-P.; Marenich A. V.; Markovich T.; Mason S.; Maurer S. A.; McLaughlin P. F.; Menger M. F. S. J.; Mewes J.-M.; Mewes S. A.; Morgante P.; Mullinax J. W.; Oosterbaan K. J.; Paran G.; Paul A. C.; Paul S. K.; Pavošević F.; Pei Z.; Prager S.; Proynov E. I.; Rák Á.; Ramos-Cordoba E.; Rana B.; Rask A. E.; Rettig A.; Richard R. M.; Rob F.; Rossomme E.; Scheele T.; Scheurer M.; Schneider M.; Sergueev N.; Sharada S. M.; Skomorowski W.; Small D. W.; Stein C. J.; Su Y.-C.; Sundstrom E. J.; Tao Z.; Thirman J.; Tornai G. J.; Tsuchimochi T.; Tubman N. M.; Veccham S. P.; Vydrov O.; Wenzel J.; Witte J.; Yamada A.; Yao K.; Yeganeh S.; Yost S. R.; Zech A.; Zhang I. Y.; Zhang X.; Zhang Y.; Zuev D.; Aspuru-Guzik A.; Bell A. T.; Besley N. A.; Bravaya K. B.; Brooks B. R.; Casanova D.; Chai J.-D.; Coriani S.; Cramer C. J.; Cserey G.; DePrince A. E.; DiStasio R. A.; Dreuw A.; Dunietz B. D.; Furlani T. R.; Goddard W. A.; Hammes-Schiffer S.; Head-Gordon T.; Hehre W. J.; Hsu C.-P.; Jagau T.-C.; Jung Y.; Klamt A.; Kong J.; Lambrecht D. S.; Liang W.; Mayhall N. J.; McCurdy C. W.; Neaton J. B.; Ochsenfeld C.; Parkhill J. A.; Peverati R.; Rassolov V. A.; Shao Y.; Slipchenko L. V.; Stauch T.; Steele R. P.; Subotnik J. E.; Thom A. J. W.; Tkatchenko A.; Truhlar D. G.; Van Voorhis T.; Wesolowski T. A.; Whaley K. B.; Woodcock H. L.; Zimmerman P. M.; Faraji S.; Gill P. M. W.; Head-Gordon M.; Herbert J. M.; Krylov A. I. Software for the Frontiers of Quantum Chemistry: An Overview of Developments in the Q-Chem 5 Package. J. Chem. Phys. 2021, 155, 084801 10.1063/5.0055522. 34470363 PMC9984241

[ref67] Iikura H.; Tsuneda T.; Yanai T.; Hirao K. A Long-Range Correction Scheme for Generalized-Gradient-Approximation Exchange Functionals. J. Chem. Phys. 2001, 115, 3540–3544. 10.1063/1.1383587.

[ref68] Grimme S.; Ehrlich S.; Goerigk L. Effect of the Damping Function in Dispersion Corrected Density Functional Theory. J. Comput. Chem. 2011, 32, 1456–1465. 10.1002/jcc.21759. 21370243

[ref69] Breneman C. M.; Wiberg K. B. Determining Atom-Centered Monopoles from Molecular Electrostatic Potentials. The Need for High Sampling Density in Formamide Conformational Analysis. J. Comput. Chem. 1990, 11, 361–373. 10.1002/jcc.540110311.

[ref70] Eastman P.; Swails J.; Chodera J. D.; McGibbon R. T.; Zhao Y.; Beauchamp K. A.; Wang L.-P.; Simmonett A. C.; Harrigan M. P.; Stern C. D.; Wiewiora R. P.; Brooks B. R.; Pande V. S. OpenMM 7: Rapid Development of High Performance Algorithms for Molecular Dynamics. PLoS Comput. Biol. 2017, 13, e1005659 10.1371/journal.pcbi.1005659. 28746339 PMC5549999

[ref71] Brock C. P.; Dunitz J. D. Temperature Dependence of Thermal Motion in Crystalline Anthracene. Acta Cryst. Sec. B 1990, 46, 795–806. 10.1107/S0108768190008382.

[ref72] Schiefer S.; Huth M.; Dobrinevski A.; Nickel B. Determination of the Crystal Structure of Substrate-Induced Pentacene Polymorphs in Fiber Structured Thin Films. J. Am. Chem. Soc. 2007, 129, 10316–10317. 10.1021/ja0730516. 17672461

[ref73] Martínez J. M.; Martínez L. Packing Optimization for Automated Generation of Complex System’s Initial Configurations for Molecular Dynamics and Docking. J. Comput. Chem. 2003, 24, 819–825. 10.1002/jcc.10216. 12692791

[ref74] Martínez L.; Andrade R.; Birgin E. G.; Martínez J. M. PACKMOL: A Package for Building Initial Configurations for Molecular Dynamics Simulations. J. Comput. Chem. 2009, 30, 2157–2164. 10.1002/jcc.21224. 19229944

[ref75] Wang Y.-S.; Wang C.-I.; Yang C.-H.; Hsu C.-P. Machine-Learned Dynamic Disorder of Electron Transfer Coupling. J. Chem. Phys. 2023, 159, 034103 10.1063/5.0155377. 37458343

[ref76] Malagoli M.; Coropceanu V.; Da Silva Filho D. A.; Brédas J. L. A Multimode Analysis of the Gas-Phase Photoelectron Spectra in Oligoacenes. J. Chem. Phys. 2004, 120, 7490–7496. 10.1063/1.1687675. 15267661

[ref77] Leggett A. J.; Chakravarty S.; Dorsey A. T.; Fisher M. P. A.; Garg A.; Zwerger W. Dynamics of the Dissipative Two-State System. Rev. Mod. Phys. 1987, 59, 1–85. 10.1103/RevModPhys.59.1.

[ref78] Jang S. J. Partially Polaron-Transformed Quantum Master Equation for Exciton and Charge Transport Dynamics. J. Chem. Phys. 2022, 157, 104107 10.1063/5.0106546. 36109233

[ref79] Makri N. Modular Path Integral Methodology for Real-Time Quantum Dynamics. J. Chem. Phys. 2018, 149, 214108 10.1063/1.5058223. 30525729

[ref80] Tang Z.; Ouyang X.; Gong Z.; Wang H.; Wu J. Extended Hierarchy Equation of Motion for the Spin-Boson Model. J. Chem. Phys. 2015, 143, 224112 10.1063/1.4936924. 26671363

[ref81] Leontyev I. V.; Stuchebrukhov A. A. Polarizable Molecular Interactions in Condensed Phase and Their Equivalent Nonpolarizable Models. J. Chem. Phys. 2014, 141, 014103 10.1063/1.4884276. 25005273 PMC4106032

